# Inferencing superspreading potential using zero-truncated negative binomial model: exemplification with COVID-19

**DOI:** 10.1186/s12874-021-01225-w

**Published:** 2021-02-10

**Authors:** Shi Zhao, Mingwang Shen, Salihu S. Musa, Zihao Guo, Jinjun Ran, Zhihang Peng, Yu Zhao, Marc K. C. Chong, Daihai He, Maggie H. Wang

**Affiliations:** 1 JC School of Public Health and Primary Care, Chinese University of Hong Kong, Hong Kong, China; 2 CUHK Shenzhen Research Institute, Shenzhen, China; 3 School of Public Health, Xi’an Jiaotong University Health Science Center, Xi’an, 710061 Shaanxi China; 4 Department of Applied Mathematics, Hong Kong Polytechnic University, Hong Kong, China; 5 Department of Mathematics, Kano University of Science and Technology, Wudil, Nigeria; 6 School of Public Health, Shanghai Jiao Tong University School of Medicine, Shanghai, China; 7 Department of Epidemiology and Biostatistics, School of Public Health, Nanjing Medical University, Nanjing, China; 8 School of Public Health and Management, Ningxia Medical University, Yinchuan, China

**Keywords:** COVID-19, Transmission, Superspreading, Heterogeneity in infectiousness, Contact tracing, Statistical inference

## Abstract

**Background:**

In infectious disease transmission dynamics, the high heterogeneity in individual infectiousness indicates that few index cases generate large numbers of secondary cases, which is commonly known as superspreading events. The heterogeneity in transmission can be measured by describing the distribution of the number of secondary cases as a negative binomial (NB) distribution with dispersion parameter, *k*. However, such inference framework usually neglects the under-ascertainment of sporadic cases, which are those without known epidemiological link and considered as independent clusters of size one, and this may potentially bias the estimates.

**Methods:**

In this study, we adopt a zero-truncated likelihood-based framework to estimate *k*. We evaluate the estimation performance by using stochastic simulations, and compare it with the baseline non-truncated version. We exemplify the analytical framework with three contact tracing datasets of COVID-19.

**Results:**

We demonstrate that the estimation bias exists when the under-ascertainment of index cases with 0 secondary case occurs, and the zero-truncated inference overcomes this problem and yields a less biased estimator of *k*. We find that the *k* of COVID-19 is inferred at 0.32 (95%CI: 0.15, 0.64), which appears slightly smaller than many previous estimates. We provide the simulation codes applying the inference framework in this study.

**Conclusions:**

The zero-truncated framework is recommended for less biased transmission heterogeneity estimates. These findings highlight the importance of individual-specific case management strategies to mitigate COVID-19 pandemic by lowering the transmission risks of potential super-spreaders with priority.

## Introduction

One of the main determinants of infectious disease dynamics is the infectiousness of its etiological agent [1–3], which is commonly quantified by the reproduction number, denoted by *R*. The *R* is defined as the average number of secondary cases generated by a typical infectious individual [4]. However, population estimates of *R* may undermine the considerable individual heterogeneity in infectiousness, as highlighted by numerous superspreading events [5–8], in which certain individuals infected unusually large numbers of secondary cases. Such heterogeneity in transmission can be estimated by describing the distribution of the number of secondary cases generated by each index case as a negative binomial (NB) distribution with dispersion parameter, *k* [9, 10]. Thus, *k* < 1 suggests that transmission is overdispersed, and hence outbreaks is likely involving superspreading events.

The heterogeneity in transmission is determined by many factors including the characteristics of host and pathogen, the setting of transmission [11], contact patterns, viability of the pathogen, and environmental components. The risk management and disease control strategies may vary and be adjusted in response to different levels of the individual heterogeneity in transmission [6, 10, 12]. As demonstrated theoretically [10], with *R* fixed, a smaller *k* results in a lower effectiveness of non-pharmaceutical interventions in controlling the epidemics, which is also discussed in [12]. Hence, methods for inferring the degree of heterogeneity in transmission, i.e., *k*, from transmission chain data have important applications in infectious disease surveillance and control.

**Fig. 1:**
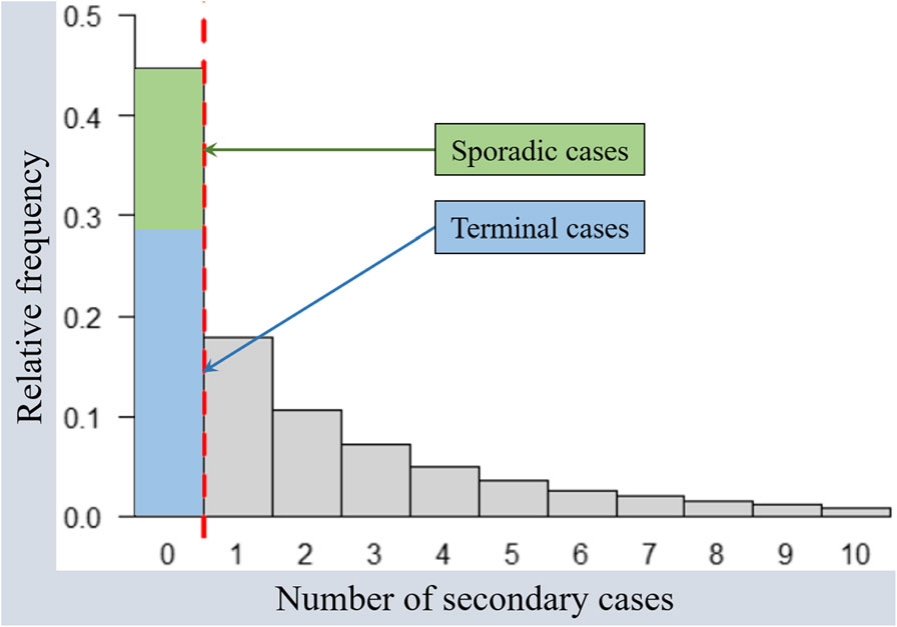
The demonstrative histogram shows the distribution of secondary cases number of each index case. For illustration, the histogram is generated by setting reproduction number, *R* = 2, and dispersion parameter, *k* = 0.5, for the negative binomial (NB) distribution. The index cases with 0 secondary case generated is divided into two sub-groups including sporadic (in green) and terminal cases (in blue)

The estimation of *k* requires real-world observations of offspring case number generated by each individual index case, which are commonly presented in the histogram as illustrated in Fig. 1. The sampling of offspring case number observations is conducted by extracting the information from the contact tracing surveillance data. However, this sampling process among contact tracing individuals might encounter sampling bias when dealing with sporadic cases. The sporadic cases, also known as single index cases, were those without known epidemiological link, and considered as independent clusters of size one [7]. Another similar type of cases is the terminal cases, and as for distinguishment, the terminal cases were those with known epidemiological links but have 0 secondary case generated. As illustrated in Fig. 1, sporadic and terminal cases are all index cases who generate 0 secondary case.

Since terminal case has its linked index case, they can be collected from the contact tracing data directly. By contrast, due to lack of epidemiological link in many situations, sporadic cases are more likely under-ascertained in contact tracing program or disease screening. Some sporadic infections with sub-clinical conditions may remain undetected after recovery. Although sporadic cases are presumed to have limited contribution to the disease transmission, under-ascertainment of these sporadic cases affects the estimation of individual heterogeneity in transmission. Specially, sporadic case is a special type of terminal case, whose index case (if any) remains unknown. In this aspect, sporadic cases need to be considered when inferring *k*. However, although sporadic cases are considered and sometimes included in the dataset used for inferring *k* [11, 13], the likelihood of under-ascertainment of such cases is usually neglected, which may potentially bias the estimates, and thus adjustment on the analytical framework may resolve this issue.

The coronavirus disease 2019 (COVID-19), caused by the severe acute respiratory syndrome coronavirus 2 (SARS-CoV-2), was firstly reported in late 2019 [14–16], then spread to over 200 countries globally in a short period of time [17, 18], and poses serious threat to public health. On one hand, similar to the previous severe acute respiratory syndrome (SARS) epidemics in 2003 [19–21], the superspreading potentials and traceable events of COVID-19 transmission were frequently reported in terms of the scale of *k* estimates [8, 11, 13, 22]. Thus, given the challenges in mitigating the ongoing COVID-19 pandemic, the inference of superspreading risk is of public health importance in informing the disease control strategic-making process. On the other hand, evidences that COVID-19 cases were under-ascertained were found in previous studies [16, 23]. The COVID-19 infection ascertainment surveillance process based on contact tracing is considered more likely to detect the cases with close contacts who are also infected, which was discussed previously [24–26]. We argue a (relatively higher) possibility of under-ascertainment of sporadic COVID-19 cases. To infer the superspreading potentials, an approach is needed to overcome the impacts due to sporadic case.

In this study, we develop a zero-truncated likelihood-based framework to estimate the individual heterogeneity in infectiousness, *k*. We demonstrate that the estimation bias exists when the under-ascertainment of index cases with 0 secondary case occurs, and the zero-truncated inference overcomes this problem and yields a less biased estimator of *k*. We exemplify the inference framework with three contact tracing datasets of COVID-19 for demonstration and comparison.

## Methods

### Heterogeneity of individual infectiousness

We consider the heterogeneity of the individual-level infectiousness as a quantifiable scale that affects the distribution of the number of offspring infectees generated by each infector. Following previous study [10], we introduce the mean number of offspring infectee generated by an infector, denoted by *r*, as a random variable from a Gamma distribution, denoted by *h*(∙), with a constant mean, *R* (> 0), and dispersion parameter, *k* (> 0). Here, *R* is the populational reproduction number that is defined as the expected (or average) number of secondary cases caused by one typical infected individual. Thus, we have *r* follows the distribution *h*(*R*, *k*). The dispersion parameter *k* governs the dispersion of the Gamma distribution.

Poisson process with rate *r*, denoted by *f*(*r*), is adopted to address the stochastic effects in transmission, and to govern the number of offspring infectees generated by each infector, which is a random variable denoted by *Z* (≥ 0) [27]. Thus, we have *Z* follows *f*(*r*) = *f*(*R*, *k*). Straightforwardly, *f*(*R*, *k*) is a negative binomial (NB) distribution with mean *R* and variance *R*∙(1 + *R*/*k*). By the definition of NB distribution, the probability that one infector generates a cluster with size *j* (≥ 1), which is denoted by **Pr**(*Z* = *j*) = *L*_*j*_, is given in Eq. (1).

1 $$ \mathbf{\Pr}\left(Z=j\right)={L}_j=\frac{\Gamma \left( kj+j-1\right)}{\Gamma (kj)\bullet \Gamma \left(j+1\right)}\bullet \frac{{\left(\frac{R}{k}\right)}^{j-1}}{{\left(1+\frac{R}{k}\right)}^{kj+j-1}}. $$

Here, Γ(∙) denotes the Gamma function.

Specially, the NB distribution *f*(∙) reduces to a Geometric distribution when *k* = 1, and it further reduces to a Poisson distribution when *k* approaches infinity. Importantly, a smaller value of *k* indicates larger heterogeneity in individual infectiousness.

### Likelihood-based inference framework and simulation scheme

We consider the two versions of the likelihood framework for the fitting and estimation. They include non- and zero-truncated versions formulated in the following Sections. We implemented the two likelihood-based inference frameworks to fit the real-world number of secondary cases observations. By fitting to the real-world observations, the dispersion parameter, *k*, can be estimated by using the maximum likelihood estimation approach. The 95% confidence intervals (95%CI) are calculated by using the profile likelihood estimation framework, which is determined by a Chi-square-distributed cutoff threshold [28, 29].

The estimating performance is evaluated by stochastic simulation with different levels of under-reporting ratio, *w*, of index cases who have 0 secondary case associated, which is introduced in the following Sections.

#### Non-truncated version

We consider observed number of offsprings from *N* infectors. Considering the infector who generates a cluster with size *j* (≥ 1), we denote the number of these infectors by *n*_*j*_. Straightforwardly, we have ∑_*j* > 1_*n*_*j*_ = *N*. Then, following the previous studies [7, 11], the likelihood of observing *n*_*j*_ clusters of size *j* is ${L_j}^{n_j}$. Thus, we construct the overall likelihood function, denoted by *ℓ*_0_, as in Eq. (2).

2 $$ {\ell}_0={\prod}_{j\ge 1}{L_j}^{n_j}. $$

#### Zero-truncated version

Same collection of notations as in the Section above are also used in this section. For all the infector who generates a cluster with size *j* > 1, we adjusted for this truncation based on the likelihood framework in Eq. (2). Namely, a zero-truncated NB-distributed likelihood framework is adopted here, and similar models with zero truncation were used previously [30]. As such, the likelihood of observing *n*_*j*_ clusters of size *j* (> 1) is ${\left(\frac{L_j}{1-{L}_1}\right)}^{n_j}$. Hence, we construct the adjusted overall likelihood function, denoted by *ℓ*_A_, as in Eq. (3).

3 $$ {\ell}_{\mathrm{A}}={\prod}_{j>1}{\left(\frac{L_j}{1-{L}_1}\right)}^{n_j}. $$

#### Simulation framework

To evaluate and compare the estimates from the non- and zero-truncated likelihood frameworks, we implemented the two inference frameworks to the random samples generated by the stochastic simulation with a known fixed *k*. For each run of the simulation, we generate the random samples with sample size at 300 from a NB distribution with *k* fixed. We estimate *k* from each 300 random samples generated. We fix *k* at 3 levels including 0.1, 0.3, or 0.9 for demonstration. For each *k*, we repeat estimating *k* for 100 runs of the simulation with different values of *R* randomly generated by a Uniform distribution ranging from 0.5 to 2.

**Fig. 2:**
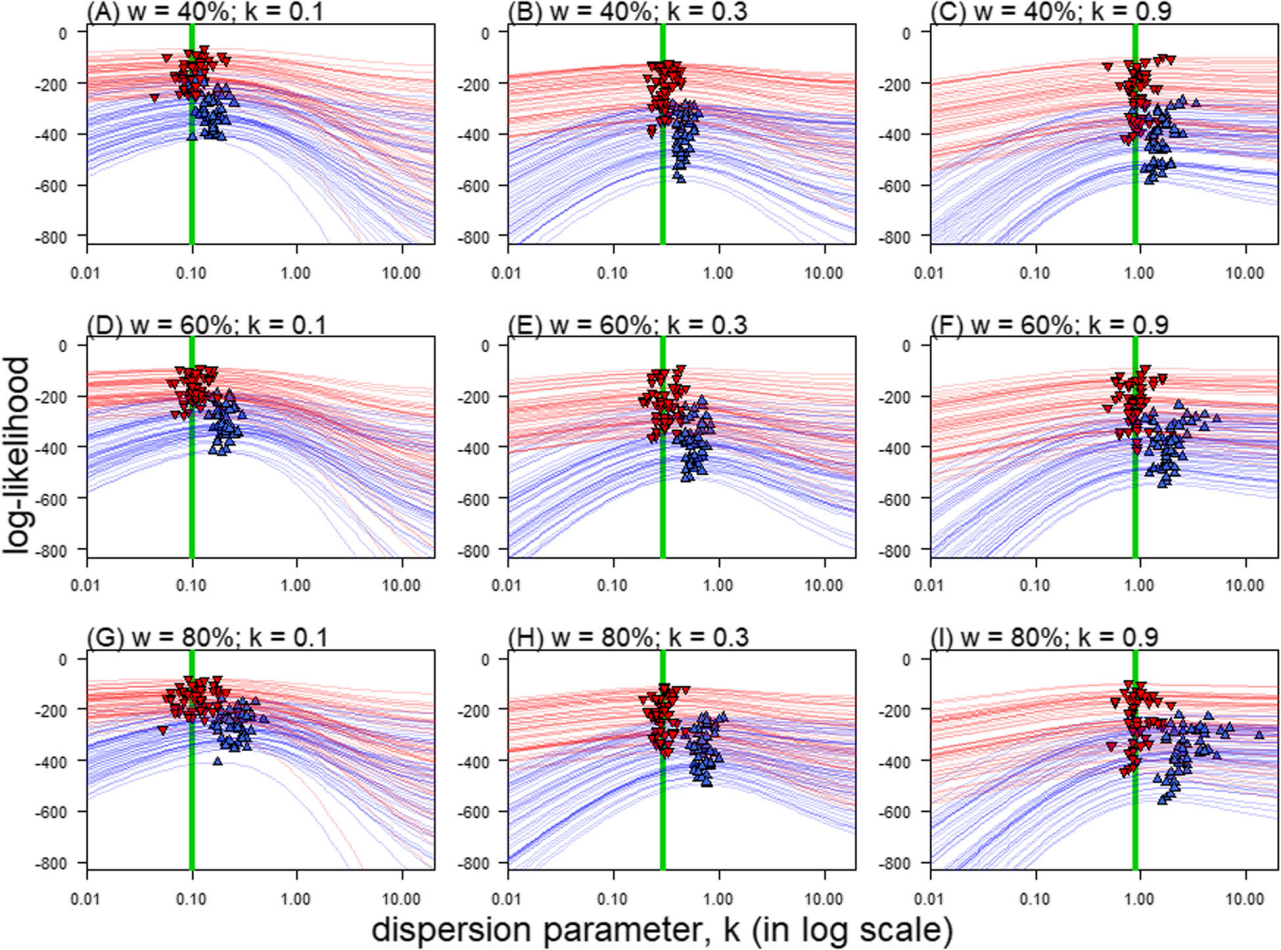
The estimation of dispersion parameter, *k*, under scenarios with different under-reporting ratios, *w*, of index cases who have 0 secondary case associated and different true values of *k*. In each panel, the curves show the log-likelihood profiles, and the triangular dots indicate the maximum likelihood estimates (MLE) of *k*. The zero-truncated version is shown in red, the non-truncated version is shown in blue, and the true values of *k* is indicated by the vertical green line

For each set of 300 samples, we remove a fraction, *w*, of samples with value 0. This setting mimics the under-reporting of index cases who have 0 secondary case associated in the real-world situation, and thus the parameter *w* is the under-reporting ratio. For demonstration, we fix *w* at 3 levels including 40, 60%, or 80%. As such, we have (3 × 3 =) 9 simulation scenarios in total, which is shown in the panel labels in Fig. 2.

The codes for simulations applying the inference framework are provided in the supplementary file of this study.

### Datasets for exemplification

For the real-world observations, we adopt three COVID-19 contact tracing datasets collected in mainland China (labelled as dataset #1), Hong Kong (labelled as dataset #2), and Tianjin, China (labelled as dataset #3) for exemplification.

One of the major differences of interest for the datasets is that dataset #1 does not include sporadic cases, but datasets #2 and #3 include sporadic cases. All datasets were collected with systematic and strict ‘inclusion-and-exclusion’ screening criteria based on plausible epidemiological evidence, and rigorous consistency-checking by several researchers independently under the supervision of a senior author.

#### Dataset #1: COVID-19 contact tracing data in mainland China

For dataset #1, we used the COVID-19 surveillance data previously published in [11], and the dataset can be accessed freely via the public respiratory https://github.com/linwangidd/covid19_transmissionPairs_China/blob/master/transmission_pairs_covid_v2.csv. The same dataset was also adopted in [12], in which the estimation of *k* was conditioned on excluding sporadic due to data unavailability.

The dataset contains 1407 transmission pairs that are identified and reconstructed according to the previous studies, governmental news release, and official situation reports in mainland China. We identified 807 infectors, and we extract the information about the number of offspring infectees generated by each infector. There are 1241 terminal cases identified from the transmission pairs. This dataset was originally collected for characterizing the epidemiological features in transmission pairs, e.g., serial interval, and thus no sporadic case is involved in this dataset.

#### Dataset #2: COVID-19 contact tracing data in Hong Kong

For dataset #2, we used the COVID-19 surveillance data previously published in [11], and the dataset can be accessed freely via the public respiratory https://github.com/dcadam/covid-19-sse/blob/master/data/transmission_pairs.csv.

The dataset contains 169 transmission pairs that are identified and reconstructed according to governmental news release, and official situation reports in Hong Kong [31, 32]. We identified 91 infectors, and we extract the information about the number of offspring infectees generated by each infector. There are 153 terminal cases identified from the transmission pairs, and 46 sporadic local cases detected in Hong Kong, which are included in the analysis.

#### Dataset #3: COVID-19 contact tracing data in Tianjin, China

For dataset #3, we used the COVID-19 surveillance data previously published in [13], and the dataset can be freely obtained from the their supplementary materials, accessed via https://www.mdpi.com/1660-4601/17/10/3705/s1. This dataset contains 36 cluster of cases including 47 COVID-19 cases, which are identified and reconstructed according to governmental news release, and official situation reports in Tianjin, China [33], and each cluster is caused by one primary case. We identified 7 infectors with 11 terminal cases associated, and 29 sporadic local cases, which are included in the analysis.

## Results and discussion

To compare the inference performance between the non- and zero-truncated frameworks, we conducted stochastic simulation to evaluate the *k* estimates from two approaches, see Fig. 2. We find that for the non-truncated framework, the estimation bias exists when the under-ascertainment of index cases with 0 secondary case occurs, and the bias increases as the level of under-ascertainment (*w*) increases. By contrast, for the zero-truncated framework, the *k* estimates are less biased for different levels of under-ascertainment (*w*).

We exemplify the inference framework with three datasets of COVID-19, and summarise the estimates in Table 1. For all datasets, our estimates of *k* using non-truncated framework are largely consistent with existing estimates in [11–13]. However, under non-truncated framework, *k* is estimated at 0.72 (95%CI: 0.63, 0.89), 0.42 (95%CI: 0.26, 0.78) and 0.22 (95%CI: 0.03, 1.15) for datasets #1, #2 and #3, respectively, which appear different from each other. We find that the *k* estimates using zero-truncated framework are smaller than those using non-truncated framework for all datasets. We note that due to the small sample size of dataset #3, the 95%CI of associated *k* estimates are relatively wide, which is considered less confident in reflecting the true scale of *k*, and thus our interpretations focus on datasets #1 and #2 in this section. Under zero-truncated framework, our estimates of *k* are 0.37 (95%CI: 0.29, 0.48) and 0.32 (95%CI: 0.15, 0.64) for datasets #1 and #2, respectively. They appear smaller than those using non-truncated framework, but are consistent with each other.

**Table 1:** The summary of the dispersion parameter, *k*, estimates of COVID-19 transmission in the existing literature and this study. The highlighted estimates are considered as main results in this study

type of dataset	data source	truncation	dispersion parameter, *k*	estimated in	sporadic case included
offspring # of each case	Dataset #1: Xu et al. [8] (*n* = 2214)	No	0.70 (0.59, 0.98)	He et al. [12]	No
			0.72 (0.63, 0.89)	this study	
		Yes	0.37 (0.29, 0.48)		
	Dataset #2: Adam et al. [11] (*n* = 290)	No	0.43 (0.29, 0.67)	Adam et al. [11]	Yes
			0.42 (0.26, 0.78)	this study	
		Yes	0.32 (0.15, 0.64)		No
	Dataset #3: Zhang et al. [13] (*n* = 47)	No	0.25 (0.13, 0.88)	Zhang et al. [13]	Yes
			0.22 (0.03, 1.15)	this study	
		Yes	0.18 (0.01, 1.79)		No
	not included in this study	No	0.58 (0.35, 1.18)	Bi et al. [34]	Yes
			range: 0.32–0.82	Lau et al. [22]	
			0.11 (0.05, 0.25)	Tariq et al. [35]	
outbreak size		not applicable	0.54 (0.01, 6.95)	Riou et al. [3]	irrelevant
			0.10 (0.05, 0.20)	Endo et al. [36]	
genome sequences			0.32 (0.13, 0.38)	Wang et al. [37]	

Notably, given dataset #2 contains some sporadic local cases, the *k* estimates under non- and zero-truncated frameworks, i.e., *k* = 0.42 and 0.32 respectively, are relatively closer than those inferred from dataset #1, i.e., *k* = 0.72 and 0.37 respectively, which does not include sporadic case. Additionally, we exclude all 46 sporadic cases in dataset #2 and repeat the estimation using non-truncated framework for comparison. We find that the *k* estimates at 0.55 (95%CI: 0.32, 1.02), which is larger than that from the zero-truncated framework. If we consider the involvement of sporadic cases as the ascertainment of these cases, which is equivalent with respect to the surveillance datasets, not including or under-ascertaining sporadic cases can be reflected by the under-reporting ratio (*w*) in the simulation scheme. Similar patterns can be found by using dataset #3, which also contains sporadic cases. Therefore, the observed difference in difference between the *k* estimates from non- and zero-truncated frameworks across datasets #1 and #2 (as well as #3, equivalently) is in line with the results shown in Fig. 2.

By screening the literature about the heterogeneity of COVID-19 infectiousness, we summarise the *k* estimates in Table 1 for comparison. Different settings and modes of transmission or contact may alter the scale of *k* [10], which could partly explain the variation of *k* estimates in different studies. For the studies considered sporadic cases, their *k* estimates are relatively smaller than those estimates without sporadic cases. The *k* estimates with zero-truncation are smaller than those without zero-truncation, and the similar patterns can also be observed in the simulation outcomes in Fig. 2. Another study using SARS-CoV-2 sequencing data [37], which appears less affected by the sporadic-case issue, estimated *k* at 0.32 (95%CI: 0.13, 0.38), and this estimate is highly consistent with our main results (highlighted in Table 1).

We find that zero-truncated framework is likely to yield a less biased *k* estimate, and the *k* of COVID-19 is inferred (slightly) smaller than many previous estimates [3, 11, 12]. With *R* fixed, a smaller *k*, which means a higher superspreading potential, leads to a lower effectiveness of population-wide non-pharmaceutical interventions in controlling the epidemics, but the individual-specific control measures are likely outperforming and more cost-effective [10, 12]. Therefore, we attach the importance of the individual-specific case management strategies for mitigate COVID-19 pandemic by lowering the transmission risks of potential super-spreaders with priority.

Regarding to the strengths, limitations, and cautiousness that need to be noted, we have the following remarks. First, as a data-driven study, the quality of estimates relies on both sophistication of the analytical framework and accuracy of real-world observations. One of the major assumptions of our model is that the accuracy of the real-world contact tracing surveillance, e.g., the three datasets in this study. Specifically, the number of offspring cases generated from each index case is presumed to be correct. As such, we acknowledge that our current modelling framework is unbale to handle the situation when there is nonnegligible inaccuracy in the surveillance data, which needs more information on patterns of the systematic error or sampling bias. Second, although we demonstrated that zero-truncated framework may yield a less biased estimator of *k*, on the ‘cost’ side, this means all observations of terminal and sporadic (if any) cases are excluded from the estimation. As consequence, more uncertainty, e.g., wider 95%CI, is likely raised under this framework. Third, more sporadic case indicated more cases without knowledge of the source of exposure, and thus implies less effectiveness of the contact tracing efforts during the surveillance of an outbreak, which is not a positive sign of disease control. Under intensive COVID-19 non-pharmaceutical interventions implemented [38], we comment that the under-ascertainment of sporadic COVID-19 case is unlikely. This indicates that for COVID-19, the under-ascertainment of sporadic case may have less impact on the *k* estimates. Fourth, despite the intensive efforts in contact tracing, the under-ascertainment issue may occur not only in sporadic cases, though more likely, but also the cases with epidemiological links in the real-world situation. A zero-truncated likelihood-based framework might also bias the estimates of *k*. However, if the sporadic cases and infectors, i.e., those with associated at least one infectee, are equally likely to be undetected, the sampling bias will vanish, and the *k* estimates will be unaffected. Fifth, our framework ignores the misreport issue, e.g., some sporadic cases are mistakenly reported as terminal cases, which needs more information on the misreporting patterns to resolve. Last, although zero-truncated framework outperforms the other in terms of the estimation unbiasedness, we remark that the original non-truncated framework is acceptable, if the effects of sporadic case can be justified at a minor scale. For instance, the situations include intensive surveillance program, and effective contact tracing when the disease have clear symptoms and strictly positive serial interval, e.g., pneumonic plague.

## Data Availability

All data used in this work were publicly available via the public data sources, please see the data Section for details. The code for simulation can be found via https://github.com/plxzpnxZBD/ZTNB_SSE_sim.
